# Expression, prognosis and preliminary investigation of the mechanism of action of ACTR6, a member of the ARPs gene family, in hepatocellular carcinoma

**DOI:** 10.3389/fmed.2025.1513233

**Published:** 2025-03-10

**Authors:** Jing Wang, Meng Song, Jinming Tang, Haoran Yue, Xiaoyang Guo, Zhan Chen, Xiaolan Shen, Mingbo Cao

**Affiliations:** ^1^Department of Gastroenterology, People’s Hospital of Zhengzhou University, Henan Provincial People's Hospital, Zhengzhou, China; ^2^Henan Provincial International Coalition Laboratory of Oncology Precision Treatment, People’s Hospital of Zhengzhou University, Zhengzhou, China; ^3^Department of Gastroenterology, Henan University People's Hospital, Henan Provincial People's Hospital, Zhengzhou, China

**Keywords:** hepatocellular carcinoma, actin-related protein family, ACTR6, cell cycle, immune infiltration

## Abstract

**Background:**

Hepatocellular carcinoma (HCC) is the third most prevalent cause of cancer-related mortality globally and the sixth most common cancer overall. It is critical to investigate new biomarkers and prognostic variables because there are currently no early diagnostic indicators. Actin-related proteins (ARPs) are involved in transcriptional regulation, chromatin remodeling, and DNA repair—all processes that have been connected to the development of cancer. However, it’s still unclear how ARPs and HCC are related.

**Methods:**

Through the examination of databases like The Cancer Genome Atlas (TCGA) and The International Cancer Genome Consortium (ICGC), we examined the variations in the expression of ARPs between the transcriptomes of normal tissue and HCC. Furthermore, univariate and multivariate Cox analysis were used to assess the prognostic effects of ARPs. The investigation of immune cell infiltration and possible functional enrichment followed. Additionally, tissue chips containing regional liver cancer specimens were used to confirm ACTR6 expression and the clinical impact of prognosis using an immunohistochemistry (IHC) test. Finally, to investigate the expression and function of ACTR6 in liver cancer cells, real-time qPCR (RT-qPCR) assays, CCK-8, clone creation, cell cycle, and transwell migration and invasion experiments were carried out.

**Results:**

We found that, in addition to ACTR3C, 17 ARPs were significantly overexpressed in HCC compared with normal tissues. In both univariate and multivariate Cox models, ACTR6 and ACTL6A were identified as potential independent risk factors for the prognosis of HCC, with ACTR6 having the lowest *p*-value. Clinical samples also confirmed this conclusion. Furthermore, ACTR6 overexpression showed a strong connection with immune cell infiltration levels and clinical and pathological factors linked to a poor prognosis. Functionally, knocking down ACTR6 inhibited cell migration and proliferation, produced a G1 cell cycle arrest, and decreased the viability of liver cancer cells.

**Conclusion:**

These findings demonstrate that ACTR6 is highly expressed in HCC and is associated with poor prognosis. In addition, ACTR6 may induce immune cell infiltration and promote hepatocarcinogenesis by regulating the cell cycle.

## Introduction

In 2020, hepatocellular carcinoma (HCC) accounted for approximately 906,000 new cases and 830,000 deaths worldwide, making it the sixth most prevalent cancer and the third highest cause of cancer-related deaths (1). Due to inadequate therapy, the 5-year overall survival of HCC patients is less than 20% (2, 3). In developing countries, the prevalence of HCC and associated cancer deaths is rising (4). More than a million individuals will pass away from HCC in 2030, according to a World Health Organization report (5). The pathophysiology of hepatocellular carcinoma (HCC) is complex, including fibrosis, liver inflammation, and abnormal hepatocyte regeneration. At the moment, surgery, chemotherapy, radiation therapy, and targeted therapy are the primary clinical therapies for liver cancer (6). Surgery is the most effective treatment for people with early-stage liver cancer when compared to other options. However, the majority of patients were detected with advanced liver cancer, losing the best opportunity for therapy since they lack diagnostic markers and show no apparent symptoms in the early stages (7). Thus, finding precise biomarkers for early diagnosis and efficacious treatment approaches is of crucial clinical importance.

Actin is widely recognized for its capacity to control membrane development, shape the cytoplasm, and guarantee the cytoskeleton’s dynamic behavior (8). Its activity is accomplished in collaboration with actin-related proteins (ARPs), a class of proteins that have structural similarities with actin. The actin superfamily, which is made up of both conventional actin and ARPs, is a highly conserved and ancient collection of proteins. Reuniting and expanding the identification and categorization of ARPs, Muller et al. (9) used a comparative genomic study of around 700 protein sequences. The ARP subfamily was split into Arp1 ~ 11, with Arp1 being the most closely linked to actin and Arp10 and Arp11 being the least (9), based on sequence consistency and similarity with typical actin. With the continuous update of understanding, ARPs family species are divided into more and more detailed. We found 18 members of the ARPs family, including ACTR1A/ACTR1B/ACTR2/ACTR3/ACTR3B/ACTR3C/ACTR5/ACTR6/ACTR8/ACTR10/ARPC1A/ARPC2/ARPC3/ARPC4/ARPC5/ARPC5L/ACTL6A/ACTL8.

Arps and actin exhibit significant sequence identity and similarity, indicating that the two proteins may share a tertiary structure that is based on a highly conserved ATPase domain—an ATP/ADP-binding pocket called the “actin fold” (9). They play a role in DNA repair, transcription control, and chromatin modification. The significance of ARPs in the development of cancer has progressively come to light recently. ACTL6A has been shown to be essential in controlling the glutathione (GSH) metabolic pathway because it increases the production of gamma-glutamylcysteine ligase catalytic subunits (GCLC). This reduces reactive oxygen species (ROS) levels and prevents iron mortality in gastric cancer cells (10). Furthermore, ACTR2 induced Wnt signaling in diffuse large B-cell lymphoma (DLBCL) and used Wnt signaling to cause DLBCL to proliferate both *in vitro* and *in vivo* (11). In addition, as early as 2020, a study on non-small cell lung cancer found that ACTR6 affects the progression of the disease by influencing the biology of tumor-associated macrophages (TAM), and served as a potential prognostic marker for lung cancer (12). In 2022, a study showed that ACTR6 is also one of the prognostic risk model genes for HER2+ breast cancer (13).

However, we do not yet understand how ARPs affect the development of liver cancer. In this study, the ARP gene family was used as a breakthrough point to discuss ACTR6’s transcriptome, genomics, immune infiltration, and prognosis in liver cancer. Immunohistochemistry (IHC) was applied to validate the expression of ACTR6 in liver cancer. The physiologic roles of ACTR6 in liver cancer cells were investigated using real-time qPCR (RT-qPCR) assays, CCK-8, clone creation, cell cycle, and transwell migration and invasion tests. Our findings will provide additional information on the significance of ARPs, especially ACTR6, in liver cancer progression.

## Methods

### Clinical specimens and data collection

The International Cancer Genome Consortium (ICGC)^1^ and TCGA^2^ provided the HCC expression and clinical prognostic data for the HCC tissue samples. There were 371 HCC tissue samples and 50 normal liver tissue samples in the TCGA dataset. Additionally, the ICGC provided information on 202 normal tissues and 243 HCC tissues.

### Analysis of survival rate of ARPs

OS was chosen as the survival outcome since the majority of HCC patients had a poor prognosis. Using a univariate Cox analysis, we eliminated ARPs that were connected to survival (*p* < 0.05). Furthermore, multivariate Cox analysis was used for analyzing the ARPs significantly related to OS and its relationship with clinicopathological factors. Besides, OS and progression-free survival (PFS) time were calculated through Kaplan–Meier plotter.^3^

### Immunity analysis

We initially analyzed 22 different immune cells in each normal and HCC sample using the CIBERSORT algorithm in R language. Next, we used TIMER, a platform accessible at https://cistrome.shinyapps.io/timer/, to evaluate ACTR6 expression in HCC, as well as its correlation with immune cell numbers and its impact on immune cell markers. The CIBERSORT and TIMER algorithms were used to assess the variations in immune cell infiltration or immunological responses between the ACTR6 high- and low-expression groups. Heatmaps with various methods showed the variations in immune cell infiltration.

### Identification of DCGs of ACTR6 in HCC

The LinkedOmics database^4^ provided a total of 8,599 co-expression genes. Then we were able to access the target gene ACTR6’s correlation gene table and correlation volcano map on the LinkFinder plate. Heatmaps of the top 50 genes that are positively and negatively correlated with ACTR6 were also acquired.

### Functional enrichment analysis

A characteristic gene list of the cell cluster, or the most important genes of ACTR6, was uploaded to the Database for Annotation, Visualization, and Integrated Discovery (DAVID, v6.8). *Homo sapiens* was chosen as the species and the official gene symbol as an identification. Ultimately, enrichment findings were acquired using Kyoto Encyclopedia of Genes and Genomes (KEGG) pathway analysis and Gene Ontology (GO) analysis. In this study, the top six outcomes were shown in increasing order of *p*-value (*p* < 0.05).

### Analysis of gene set variation (GSVA)

Genes associated with the cell cycle were compiled using the AmiGO 2 portal.^5^ Every HCC sample’s functional enrichment score was determined using the provided package (R environment) and default settings. Using the pheatmap package (R environment), a heatmap of the enrichment results was created. Pearson correlation analysis was used to ascertain the relationship between ACTR6 and cell cycle.

### Cell culture and human tissues

Huh7, HepG2, and Hep3B were human HCC cell lines obtained from the Chinese Academy of Sciences Cell Bank. These cell lines were maintained in DMEM (Invitrogen, # 11965-118; CA) with 1% penicillin–streptomycin and 10% FBS added. We acquired the human normal liver cell line Thle-2 from Keycell Biotechnology (Wuhan, China). The Thle-2 cells were kept alive in a unique growth medium that Keycell Biotechnology supplied. The cells were cultivated at 37°C with 5% CO2 in a humidified incubator. Every 3 months, the absence of mycoplasma infection was confirmed in all cell lines. Shanghai Outdo Biotech Co., Ltd. provided the formalin-fixed paraffin-embedded HCC cancer tissue microarrays (HLivH180Su30).

### Antibodies (abs)

The rabbit polyclonal antibody against ACTR6 (Catalog # PA5-58453) was from Thermo Fisher Scientific (Waltham, MA). ImmPRESS Universal Polymer Reagent (Horse Anti-Mouse/Rabbit IgG) was from Vector Laboratories (S.F, CA).

### SiRNA oligos

SiRNAs were transfected using the lipofectamine 3000 Transfection Kit (ThermoFisher Scientific, #L3000-015) after being obtained from Hippobio in Huzhou, China. CCGAGAUAAUCCUUCCGAAUUTT (ACTR6 siRNA1), GCCUGACUUCAGUACAAUUAATT (ACTR6 siRNA2), and GCACAUAGGUAUUUCCGAGAUTT (ACTR6 siRNA3) were the siRNA sequences aimed against ACTR6.

### Quantitative real-time PCR (qPCR)

Following the manufacturer’s instructions, total RNA was isolated from cultivated cells using the FastPure^®^ Cell/Tissue Total RNA Isolation Kit V2 (Vazyme, # RC112; Nanjing, China). Using a StepOnePlusTM Real-Time PCR System (ThermoFisher Scientific), 15 ng of cDNA that had been generated from 900 ng of total RNA using the PrimScriptTM RT reagent Kit with gDNA Eraser (TaKaRa, #RR047A; Dalian, China) was exposed to qPCR. The relative gene expression levels were normalized against β-actin using the 2^−ΔΔCT^ technique. Supplementary Table S1 has a listing of primer sequences.

### Cell viability assay

Five thousand cells per well of 96-well plates were used to plate Huh7 cells transfected with NC or siACTR6. At the 24-, 48-, and 72-h mark, CCK-8 (DOJINDO, # CK04, Japan) was added, and the mixture was incubated for a further 2 h. Then, the plates were assayed by testing the absorbance at 450 nm.

### Colony forming assay

One thousand transfected cells were cultivated for 10–14 days at 37°C with 5% CO2 after being seeded onto 6-well plates. After discarding all liquids, the samples were fixed for 30 min at room temperature in 4% paraformaldehyde (Servicebio, #G1101; Wuhan, China), and then stained with crystal violet (Beyotime, #C0121; Beijing, China). Bio-Rad’s Quantity One^®^ software was used to take counts and photos.

### Transwell migration assay

Serum-free media was used to sustain the cells after they were planted into the top chambers of 24-well transwell inserts (8 μm, Corning, #3422, 5 × 104 cells/well). The medium was added to the bottom chambers along with 10% FBS. Following a 16-h incubation period, the cells grown in inserts were fixed for 30 min using 4% paraformaldehyde (Servicebio, #G1101; Wuhan) and stained for 8 min with crystal violet dye solution (Beyotime, #C0121; Beijing, China). Using a cotton-tipped swab, the non-migrated cells on the membranes’ top surface were scrubbed away. Before being quantified using the ImageJ program, cells that moved to the membranes’ bottom surface were further photographed using an Olympus IX73 Fluorescence Microscope System.

### Transwell invasion assay

Using inserts that had been pre-coated with a suitable quantity of Matrigel (BD Biosciences, # 356234; Franklin Lakes, NJ) combined with precooled media at a ratio of 1:8, cell invasion was measured. The cells were cultured in serum-free media and seeded into the top chambers of 24-well transwell inserts (8 μm, Corning, #3422, 1 × 105 cells/well). The next experiment followed a similar protocol to the transwell migration test previously reported.

### Immunohistochemistry assay and evaluation

The anti-ACTR6 antibody (dilution ratio, 1:200) was incubated for 10 h at 4°C on HCC and nearby tissues. After that, the tissues were treated for 30 min at 37°C with Horse Anti-Mouse/Rabbit IgG. The samples were then treated with DAB Peroxidase Substrate Kit for the reaction, and hematoxylin was used as a counterstain. Two different, skilled pathologists evaluated each IHC staining; they were unaware of the patient’s clinical status or diagnosis beforehand. ACTR6 staining score, including staining density and intensity. The scoring methods were as follows: (1) The percentage of staining positive cells was divided into 5 grades, namely <1%, 1–25%, 26–50%, 51–75%, and 76–100%, which were defined as 0, 1, 2, 3, and 4 points respectively; (2) The staining intensity was divided into 4 grades: negative staining (no staining, 0 points), weak staining intensity (light brown, 1 point), medium staining intensity (brown, 2 points), and strong staining intensity (dark brown, 3 points). (3) Immunoreactive score (IRS) = percentage of positive cells × staining intensity score, with a total score of 0 ~ 12, in which 0 ~ 5 is defined as low expression, and 6 ~ 12 is defined as high expression.

### Statistics

For all statistical analyses, SPSS 25.0 (SPSS Software, Chicago, United States), GraphPad Prism 9and R v 4.3.2^6^ were used. For statistical analysis, the Student’s t test, Mann–Whitney test, one-way analysis of variance (ANOVA) test, or Pearson correlation coefficient were used. In addition, we investigated the connection between genes and illness prognosis using COX analysis.

## Results

### Transcriptional levels of ARPs family between HCC and normal tissues

**Figure 1:**
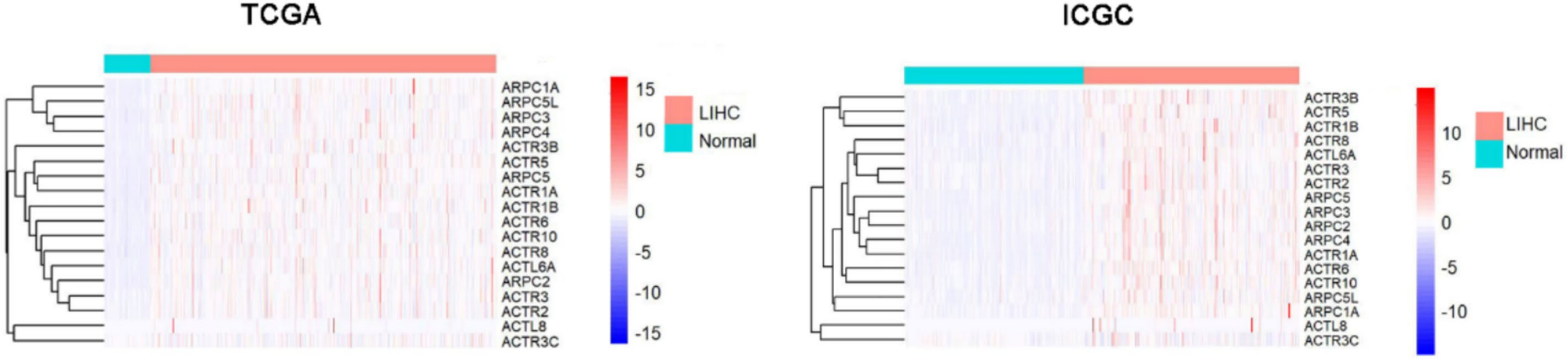
ARPs family transcriptional levels in TCGA and ICGC comparing HCC to normal tissues. HCC, hepatocellular carcinoma; ICGC, The International Cancer Genome Consortium; ARP, actin-related proteins; TCGA, The Cancer Genome Atlas.

ARPC3/ARPC5L/ARPC5/ARPC2/ARPC4/ARPC1A/ACTL8/ACTL6A/ACTR6/ACTR5/ACTR8/ACTR1B/ACTR3/ACTR10/ACTR3B/ACTR2/ACTR1A were significantly overexpressed in HCC tissues more than in normal liver tissues, according to a combination of the analysis results of The Cancer Genome Atlas (TCGA) and International Cancer Genome Consortium (ICGC) databases (Figure 1; Supplementary Figure S1).

### Prognostic value and independent prognostic value of ARPs in HCC patients

**Figure 2:**
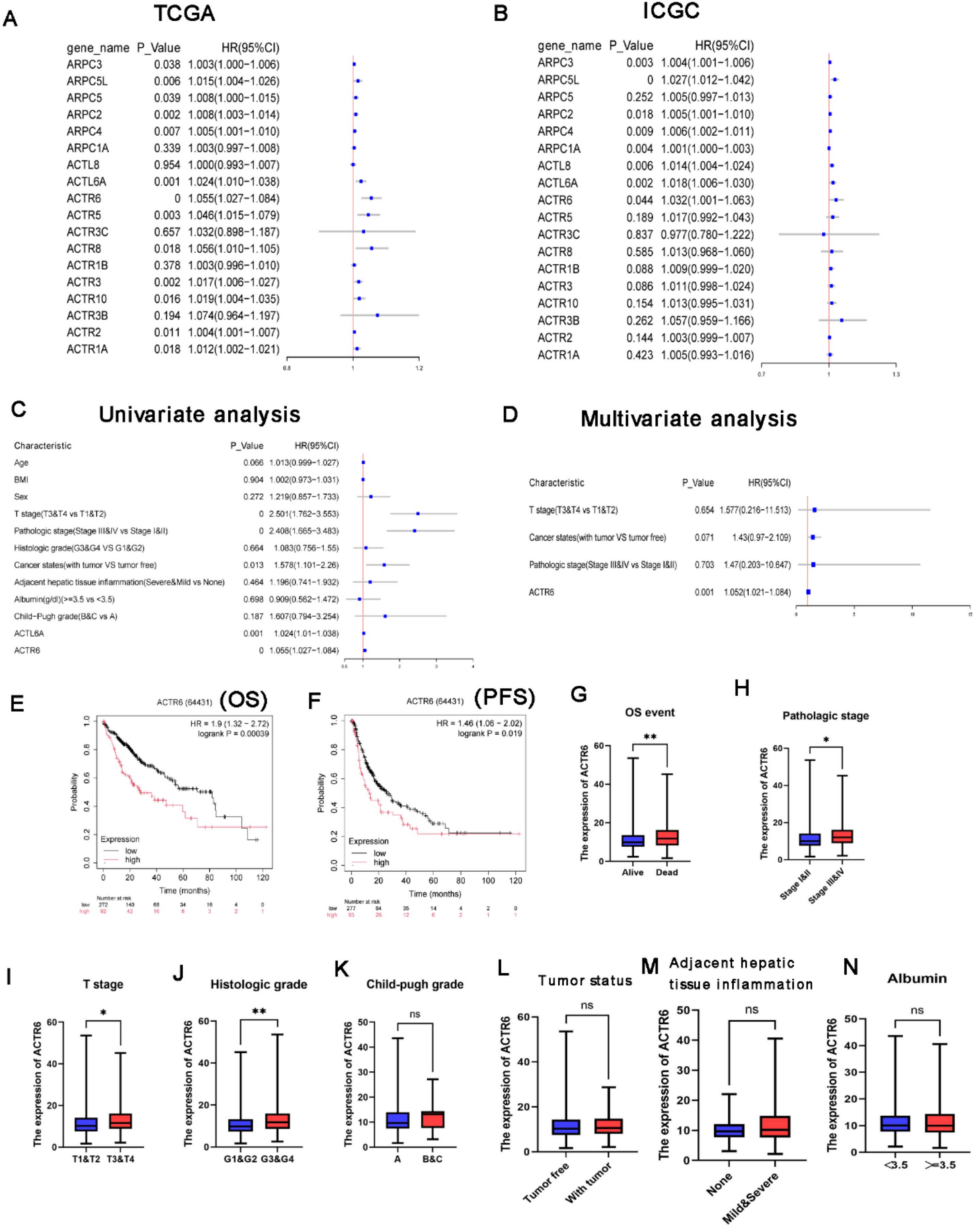
Value of distinct ARPs mRNA expressions for prognosis in hepatocellular cancer. **(A,B)** In HCC patients, there was a correlation between the mRNA expression level of ARPs and OS. ACTR6 and patient OS were analyzed using univariate **(C)** and multivariate **(D)** Cox regression. The association between ACTR6 and several clinicopathological variables as determined by the Student’s t test. In patients with HCC, shorter overall **(E)** and progression-free survival **(F)** times were associated with ACTR6 mRNA expression. Events related to OS **(G)**, pathologic stage **(H)**, T stage **(I)**, histologic grade **(J)**, child-pugh grade **(K)**, tumor states **(L)**, inflammation of the surrounding hepatic tissue **(M)**, and albumin **(N)**.

Further investigation into the relationship between ARPs expression and patient OS in the TCGA and ICGC databases revealed that, according to Univariate analysis, shorter OS in HCC was correlated with higher levels of ARPC2/ARPC3/ARPC4/ARPC5L/ACTR6/ACTL6A mRNA expression (Figures 2A,B), as well as with T stage, pathological stage, and tumor status (Figure 2C). Further multivariate analysis revealed that longer OS in HCC patients was correlated with higher ACTL6A/ACTR6 mRNA expression (Supplementary Figure S2). This suggests that ACTL6A/ACTR6 may be independent risk factors for predicting the prognosis of HCC patients, with ACTR6 showing the lowest *p*-value of 0.001 in multivariate Cox analysis (Figure 2D). Additionally, we investigated the survival data for ACTR6 expression in HCC using a Kaplan–Meier plotter (Figures 2E,F).

### The association between ACTR6 expression and clinicopathological factors in patients with HCC

Based on the research mentioned above, it was shown that ACTR6 may be used as an independent prognostic factor to predict the prognosis of patients with HCC. After examining the relationship between ACTR6 expression and clinicopathological features of HCC patients, we found that ACTR6 expression was higher in deceased patients (Figure 2G) and higher in pathological stages (Figure 2H), T stages (Figure 2I) and histological grades (Figure 2J). Nevertheless, increased child-pugh grade (Figure 2K), tumor status (Figure 2L), surrounding hepatic tissue inflammation (Figure 2M), and albumin (Figure 2N) were not linked to increased expression of ACTR6.

### Functional enrichment analysis of DCGs of ACTR6 in HCC

**Figure 3:**
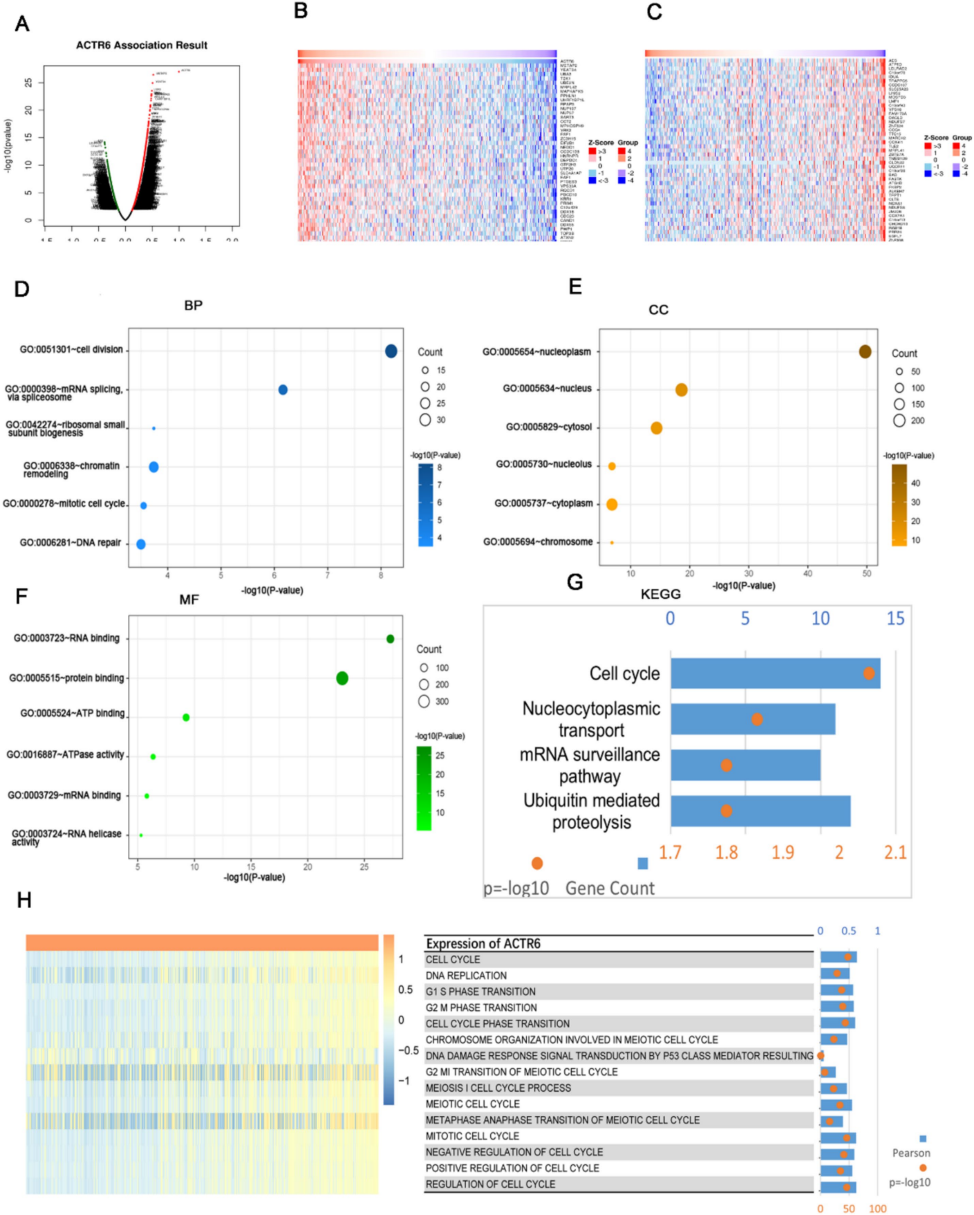
Analysis of functional enrichment of ACTR6 genes that are differently co-expressed in HCC. **(A)** Volcano plots of ACTR6 genes that are differently co-expressed. The top 50 were shown both negatively **(C)** and positively **(B)** on the heatmap. In HCC, ACTR6 is intimately related to the cell cycle. **(D–F)** The TCGA database links ACTR6 to biological processes (BP), cellular components (CC), and molecular functions (MF). **(G)** ACTR6 pathway analysis from the Kyoto Encyclopedia of Genes and Genomes (KEGG) in the TCGC database. **(H)** Pearson connection between cell cycle (GSVA) and ACTR6. The *R*-value was expressed by the band’s width.

We selected 8,599 DCGs from the LinkedOmics database, including 5,969 positively correlated and 2,630 negatively correlated genes, in order to investigate the possible mechanism by which ACTR6 operates in HCC (Figure 3A). The top 50 DCGs that are correlated with ACTR6 were shown on heatmaps (Figures 3B,C). To investigate the biological activities associated with ACTR6, the TCGA databases’ most closely related genes were eliminated by Pearson correlation analysis (|R| > 0.4, *p* < 0.05). In order to examine the important biological processes and functions, these linked genes were selected for GO and KEGG enrichment analysis. The GO analysis found that the DCGs were mostly involved in the cell division, mRNA splicing, and ribosomal small subunit biogenesis at the biological process level (Figure 3D). The cellular component enrichment study indicates that the most enriched category is nucleoplasm (Figure 3E). The most enriched categories at the molecular function level were RNA binding and protein binding (Figure 3F). The KEGG enrichment analysis showed that the cell cycle pathway was most significantly regulated by DCGs (Figure 3G). These findings suggested that ACTR6 on HCC probably plays an essential role in cell cycle.

### Tumor cell ACTR6 is positively correlated with most processes of cell cycle

The TCGA databases’ gene set variation analysis was utilized to calculate the cell cycle’s enrichment score. The enrichment score and ACTR6 expression were connected, and the results indicated that ACTR6 expression was positively correlated with most cell cycle events, with the exception of damage response signal transduction via p53 class mediator (Figure 3H). These findings revealed a connection between cell cycle and ACTR6.

### The relationship of ACTR6 expression level with immune infiltration in patients with liver cancer

**Figure 4:**
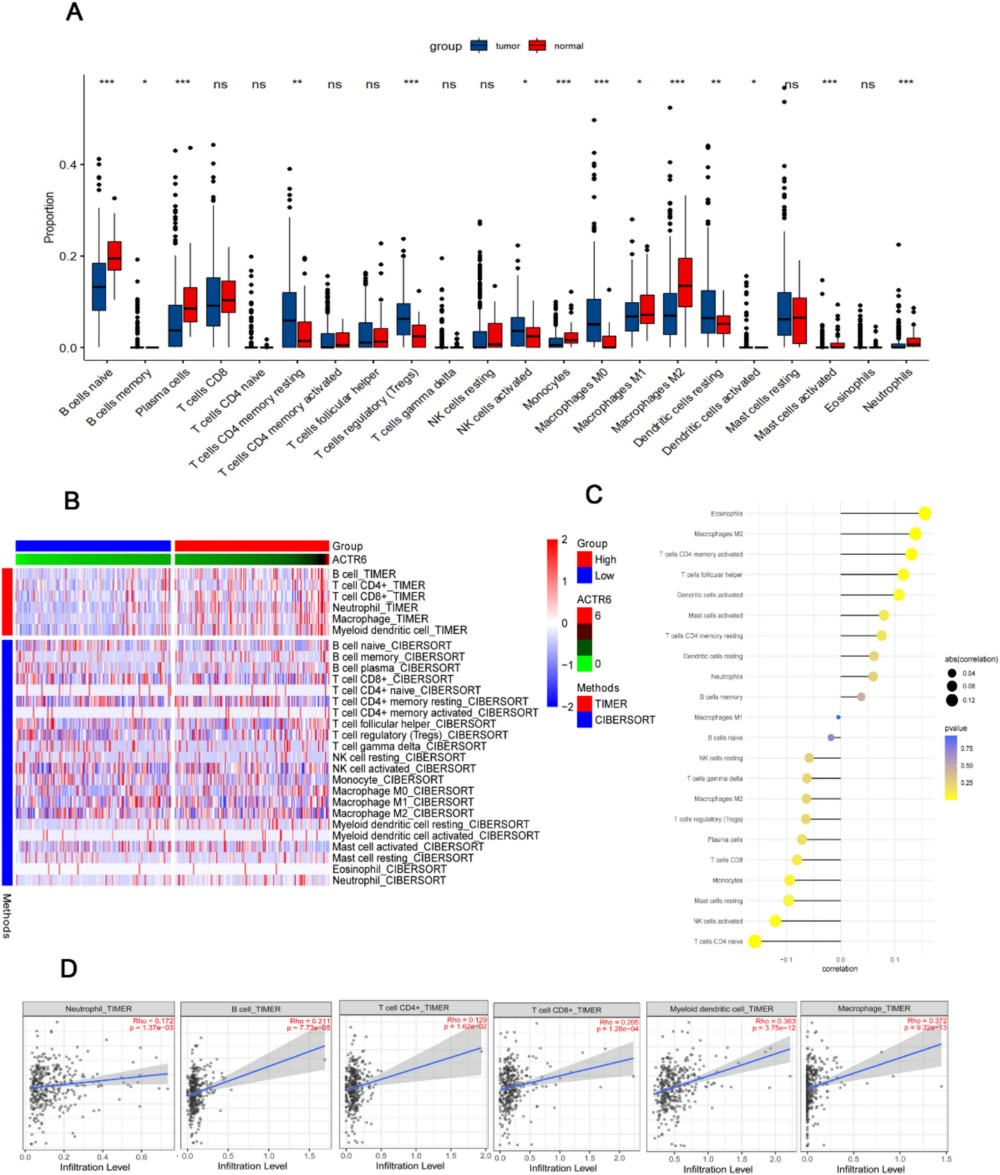
Relationship between immune infiltration in HCC and ACTR6 mRNA expression. **(A)** The comparison of estimated fractions of 22 immune cells between the normal and HCC patients. **(B)** The heat map illustrates how immune cells infiltrate differently in HCC samples with high and low ACTR6 expression. **(C)** The Lollipop graph illustrates the relationship between immune cells and ACTR6 expression. **(D)** The TIMER online analysis revealed a favorable correlation between immune cell expression and ACTR6 expression levels.

The development and spread of HCC are significantly influenced by immune infiltrations (14). We assessed the degrees of immune cell infiltration in both normal and HCC patients using the CIBERSORT and TIMER algorithms. The findings demonstrated that HCC patients had a greater degree of immune cell infiltration than did healthy individuals (Figure 4A). Furthermore, among patients with HCC, the group exhibiting high expression of ACTR6 had a greater amount of immune cell infiltration (Figure 4B). ACTR6 was positively correlated with Eosinophils, Macrophages M0, T cells CD4 memory activated, etc., and negatively correlated with T cells CD4 naive, NK cells activated, etc. (Figure 4C). Additionally, we employed TIMER online analysis to examine the correlation between ACTR6 expression and six types of immunological infiltrations (Figure 4D). To better understand the relationship between ACTR6 expression and immune infiltration, we also used the TIMER database to investigate the relationship between ACTR6 and immune cell markers. Following purity-based correction, we found that most immune cell indicators positively correlated with ACTR6 expression (Table 1).

**Table 1:** Analysis of the correlation between ACTR6 and related immune cell genes and markers in TIMER.

Description	Gene markers	ACTR6			
		Purity		None	
		Cor	*p*	Cor	*p*
B cell	CD19	0.192685	***	0.193888	***
	CD79A	0.09444	0.079827	0.076664	0.140524
Monocyte	CD86	0.392374	***	0.318948	***
T cell (general)	CD3D	0.158044	**	0.127261	*
	CD3E	0.218304	***	0.17224	***
	CD2	0.182701	***	0.139423	**
TAM	CCL2	0.241741	***	0.196835	***
	CD68	0.338994	***	0.296812	***
	IL10	0.342886	***	0.299218	***
M1	NOS2	0.211114	***	0.206415	***
	IRF5	0.369551	***	0.377102	***
	PTGS2	0.355037	***	0.281689	***
M2	CD163	0.321635	***	0.268725	***
	VSIG4	0.285164	***	0.227841	***
	MS4A4A	0.296369	***	0.23676	***
Neutrophils	CEACAM8	0.102313	0.057633	0.08704	0.094126
	ITGAM	0.379607	***	0.32761	***
	CCR7	0.137846	*	0.115018	*
Natural killer cell	KIR2DL1	0.023314	0.666093	0.051379	0.32367
	KIR2DL3	0.17147	**	0.155669	**
	KIR2DL4	0.140983	**	0.134195	**
	KIR3DL1	0.143188	**	0.137327	**
	KIR3DL2	0.107088	*	0.091035	0.079915
	KIR3DL3	0.066917	0.215048	0.102335	*
	KIR2DS4	0.089281	0.0978	0.090521	0.08164
Dendritic cell	HLA-DPB1	0.199509	***	0.16376	**
	HLA-DQB1	0.105234	0.050823	0.080124	0.123384
	HLA-DRA	0.284756	***	0.240638	***
	CD1C	0.212096	***	0.184169	***
	NRP1	0.497254	***	0.488839	***
	ITGAX	0.370632	***	0.317812	***
Th1	TBX21	0.149547	**	0.124282	*
	STAT4	0.187802	***	0.171704	***
	STAT1	0.436577	***	0.430249	***
	IFNG	0.210708	***	0.178557	***
	TNF	0.309106	***	0.262698	***
Th2	GATA3	0.230782	***	0.176471	***
	STAT6	0.428644	***	0.453155	***
	STAT5A	0.362736	***	0.346094	***
	IL13	0.105611	*	0.106375	*
Tfh	BCL6	0.38906	***	0.394642	***
	IL21	0.123442	*	0.091167	0.079478
Th17	STAT3	0.36847	***	0.363055	***
	IL17A	0.164181	**	0.170844	***
Treg	FOXP3	0.290511	***	0.274022	***
	CCR8	0.476939	***	0.431116	***
	STAT5B	0.531094	***	0.536735	***
	TGFB1	0.228634	***	0.199066	***
T cell exhaustion	PDCD1	0.162778	**	0.156384	**
	CTLA4	0.22184	***	0.190123	***
	LAG3	0.094862	0.078482	0.092361	0.075612
	HAVCR2	0.356368	***	0.283981	***
	GZMB	0.07015	0.193652	0.076324	0.142246

### Expression validation of ACTR6 in liver cancer

**Figure 5:**
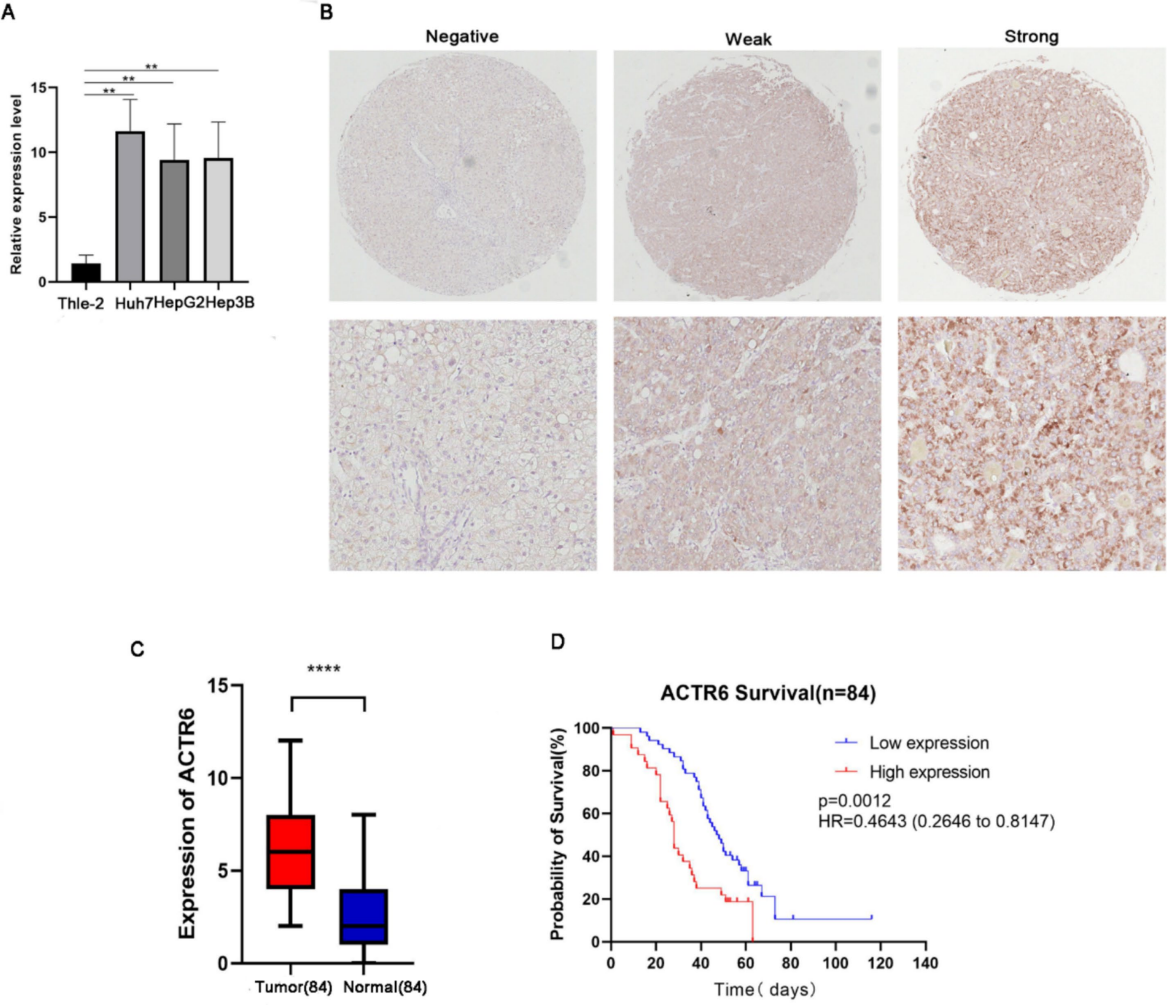
The expression of ACTR6 in normal liver cell line and liver cancer cell line Thle-2 was detected by RT-PCR **(A)**. A comparison of paired neighboring normal tissues and ACTR6 expression in formalin-fixed paraffin-embedded (FFPE) hepatocellular carcinoma (*n* = 84 physiologically independent samples) using representative microphotographs **(B)** and a student’s *t* test analysis **(C)**. **(D)** Kaplan–Meier analysis of the overall survival probability of 84 HCC patients, stratified by ACTR6 expression.

Through RT-qPCR we found that the expression level of ACTR6 in the three HCC cell lines was higher than that in normal liver cells (Figure 5A) Furthermore, to confirm the expression of ACTR6 in hepatocellular carcinoma and its prognostic effect, we used immunohistochemical methods to detect the protein expression of ACTR6 in 90 pairs (six pairs of which were lost) of formalin-fixed paraffin-embedded (FFPE) liver tissue samples (Figure 5B). It is determined that hepatocellular carcinoma has greater levels of ACTR6 protein expression (Figure 5C). However, the high expression of ACTR6 does not appear to be related to various clinicopathological factors (Table 2), which may be due to insufficient sample size. Further survival analysis showed that the higher the expression of ACTR6 in liver cancer tissues, the shorter the patients’ OS (Figure 5D). In addition, multivariate Cox regression analysis also showed that high expression of ACTR6 was correlated with OS (Table 3). Taken together, these results suggest that ACTR6 expression is up-regulated in HCC, and its high expression is associated with poor prognosis in HCC patients.

**Table 2:** Relationship between ACTR6 expression and clinicopathological features of hepatocellular carcinoma patients.

Characteristics	ACTR6 expressions		*p* value^a^
	Low	High	
Sex			
Male	45	28	0.899
Female	7	4	
Age			
≤60	44	19	0.009
>60	8	13	
T stage			
T1-2	17	15	0.194
T3-4	35	17	
ACJJ Stage^b^			
Stage l-ll	18	15	0.264
Stage ll-V	34	17	
Liver cirrhosis			
Positive	34	20	0.789
Negative	18	12	

a Chi-square test; a *p* value less than 0.05 was considered statistically significant.

b AJCC, American Joint Committee on Cancer.

**Table 3:** Multifactorial analysis affecting the overall survival of patients with hepatocellular carcinoma.

Characteristic	Multivariate analysis		
	HR	CI (95%)	*p* value
Age (≤60 years vs. >60 years)	1.856	1.059–3.252	0.031
Pathologic stage (Stage III&IV vs. Stage I&II)	1.925	1.130–3.279	0.016
T stage (T3&T4 vs. T1&T2)	1.389	0.821–2.348	0.220
ACTR6 expression (high vs. low)	1.828	1.057–3.163	0.031

### Functional analysis of ACTR6 in liver cancer cell

**Figure 6:**
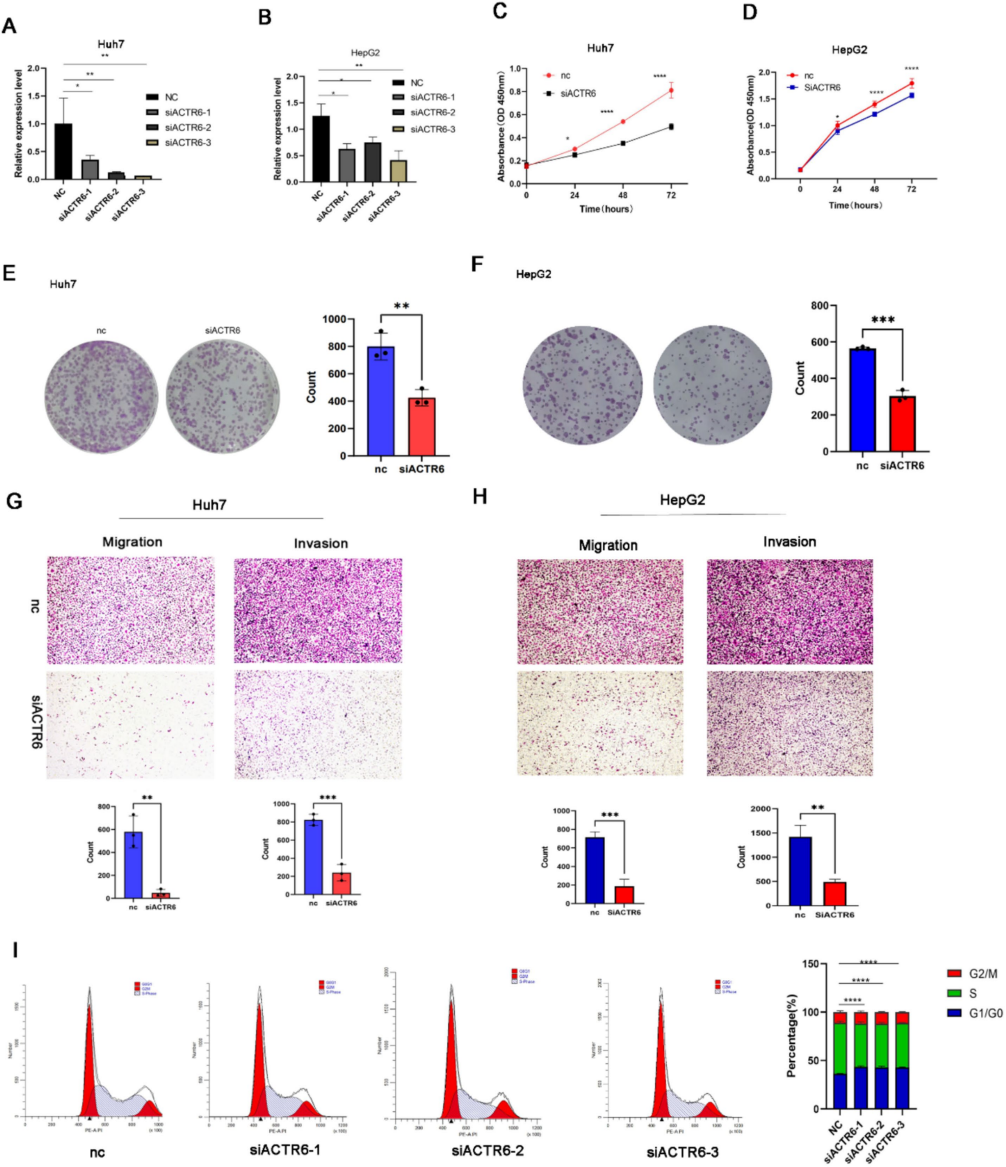
ACTR6 knockdown inhibited cell migration and proliferation. SiRNA suppression of ACTR6 **(A,B)** reduced proliferation **(C,D)** and clonal formation **(E,F)** as well as migration and invasion **(G,H)** of Huh7 and HepG2 cells. Scale bars, 200 μm (cell migration and invasion assay). Using the ImageJ program, the numbers of clonal formation, migratory, and invasive cells were quantified. After 24 h, Huh7 cells had a G1 cell cycle arrest due to ACTR6 knockdown **(I)**.

Two significant indicators of the development of cancer are the modification of the extracellular matrix and dysregulated cell cycle (14). We tried to ascertain ACTR6’s function in controlling cell migration and proliferation based on the GSVA results. We used three separate siRNAs to knock down ACTR6 in Huh7 and HepG2 cells in order to functionally investigate the function of this gene in controlling the development of hepatocellular carcinoma (Figures 6A,B). We discovered that ACTR6 knockdown markedly reduced cell proliferation through investigations on clonal formation and cell proliferation (Figures 6C–F). Furthermore, transwell experiment demonstrated a substantial reduction in cell migration following ACTR6 siRNA transfection (Figures 6G,H). Ultimately, we demonstrated by flow cytometry that ACTR6 knockdown resulted in G1 cell cycle arrest (Figure 6I).

## Discussion

Many factors, such as obesity, type 2 diabetes, metabolic syndrome, and nonalcoholic fatty liver disease (NAFLD), contribute to the development of HCC. It is still among the most deadly malignant tumors in the world (15). Abnormal malignant cell activities such as proliferation, migration, invasion, and autophagy are associated with the formation and progression of HCC (16, 17).

ARPs are necessary for the function of chromatin remodeling complexes (18). Previous studies have shown that Arp4–Arp9 are found in chromatin remodeling and histone modification complexes (19). They play a role in DNA repair, transcription control, and chromatin modification (20). For instance, ACTL6A, also referred to as Arp4, is involved in both the activation and repression of transcription from genes (21–24). ACTR5 and ACTR8, also referred to as Arp5 and Arp8, are particular INO80 complex components that are preserved. Both of them are mainly found within the INO80 complex in yeast. They are essential to the enzymatic activity of INO80 (25). Unlike other nuclear Arps, which are primarily associated with transcriptional activation, ACTR6, also known as Arp6, is required to maintain gene silence in heterochromatin. Research has demonstrated that human and chicken ACTR6 directly interact with heterochromatin protein 1 (HP1) *in vitro* (26, 27). Furthermore, the vertebrate ACTR6 is necessary for the SRCAP complex to function (18). The histone variation H2A.Z is incorporated into nucleosomes at promoter areas by the SRCAP complex. Via transcriptional control and chromosomal architecture, H2A.Z is linked to several physiological processes, such as stem cell maintenance, cellular proliferation, and stress response (27–29). It also plays significant roles in epigenetic regulation and chromosome architecture.

However, the involvement of ARP family proteins in HCC has not been completely investigated. We are in charge of this investigation of the expression, mutation, and prognostic significance of different ARP family members in HCC. After analyzing the TCGA and ICGC datasets, we discovered that patients with HCC had highly overexpressed versions of 17 ARPs. A favorable correlation was found between short OS and overexpression of ARPC2/ARPC3/ARPC4/ARPC5L/ACTR6/ACTL6A. Additional multivariate analysis showed that ACTR6/ACTL6A may function as separate risk variables for the prognosis of patients with colorectal cancer. We confirmed the significant expression of ACTR6 in liver cancer cells and liver tumor tissues, as well as its impact on the prognosis of liver cancer patients, using cell assays and immunohistochemistry analysis. Next, we looked at ACTR6’s involvement in liver cancer in more detail. On the one hand, aberrant proliferation of malignant tumors is primarily caused by dysregulation of the cell cycle. In order to estimate ACTR6’s biological role, we used GO, KEGG, and GSVA analysis. This revealed that ACTR6 may control the cell cycle, which in turn controls cell migration and proliferation. The next functional tests confirmed that ACTR6 knockdown inhibited cell proliferation by causing a G1/S cell cycle arrest as well as limiting cell migration. Cancer is characterized by persistent proliferative signaling, which promotes unending and excessive cycles of cell division. It has recently become clear that, rather than causing unchecked cell cycle advancement, these mutations that impede cell cycle exit and prevent apoptosis are what propel this uncontrollably dividing cell. These include mutations in the signaling pathways that trigger cell cycle exit or encourage entrance into the S phase (30), although they are significantly less common in the pathways that obstruct mitotic entry (31–34) and exit (35–39). On the other hand, it is thought that a crucial factor contributing to the development of HCC is the chronic inflammatory response (40–42). Our research revealed a significant association between ACTR6 and immune cells, particularly in macrophage and dendritic cells. Through the upregulation of proinflammatory cytokines and immunological checkpoints, immune cells aid in the development of tumors by immune escape (43).

Due to the limitations of our work, additional research is required to explore the possible molecular mechanism behind the carcinogenesis of ARP family proteins. However, we present the first information on the ARPprotein family’s differential expression in HCC, specifically in relation to ACTR6, as well as its possible diagnostic and prognostic significance. The expression of ACTR6 is strongly correlated with a bad prognosis in HCC. It can be explored as a potential target for HCC treatment and prognostic biomarkers. Preliminary mechanistic investigations have indicated that it increases HCC formation and progression via regulating cell cycle and immune cell infiltration. In the future, more *in vivo* experiments should be done to verify the expression of ACTR6 and further explore the specific mechanism of ACTR6 promoting the cell cycle conversion of liver cancer.

## Data Availability

The original contributions presented in the study are included in the article/Supplementary material, further inquiries can be directed to the corresponding author.

## Footnotes

^1^ https://daco.icgc.org/

^2^ https://cancergenome.nih.gov/

^3^ http://kmplot.com/analysis/

^4^ https://www.linkedomics.org/login.php

^5^http://amigo.geneontolo gy.org/amigo

^6^ https://www.r-project.org/
