# Supplementary material for: Expression, prognosis and preliminary investigation of the mechanism of action of ACTR6, a member of the ARPs gene family, in hepatocellular carcinoma
Source: Front Med (Lausanne). 2025 Mar 10;12:1513233. doi: 10.3389/fmed.2025.1513233 (PMC11931126; doi:10.3389/fmed.2025.1513233)
Supplement: Supplementary file 1 [file Table_1.DOCX]

**Supplementary Table 1**: Primer sequences of quantitative real-time PCR.

| Gene | Sequences 5'-3' |
| --- | --- |
| ACTR6 | (Forward) CGACCTTAGTGCTGGATAATGGA |
|  | (Reverse) CCCAAACTTGTCTCTGAACATCC |
| **β**-actin | (Forward) CTGGGACGACATGGAGAAAA |
|  | (Reverse) AAGGAAGGCTGGAAGAGTGC |
|  |  |
|  |  |
|  |  |
|  |  |
