# Supplementary figures and images for: Expression, prognosis and preliminary investigation of the mechanism of action of ACTR6, a member of the ARPs gene family, in hepatocellular carcinoma
Source: Front Med (Lausanne). 2025 Mar 10;12:1513233. doi: 10.3389/fmed.2025.1513233 (PMC11931126; doi:10.3389/fmed.2025.1513233)

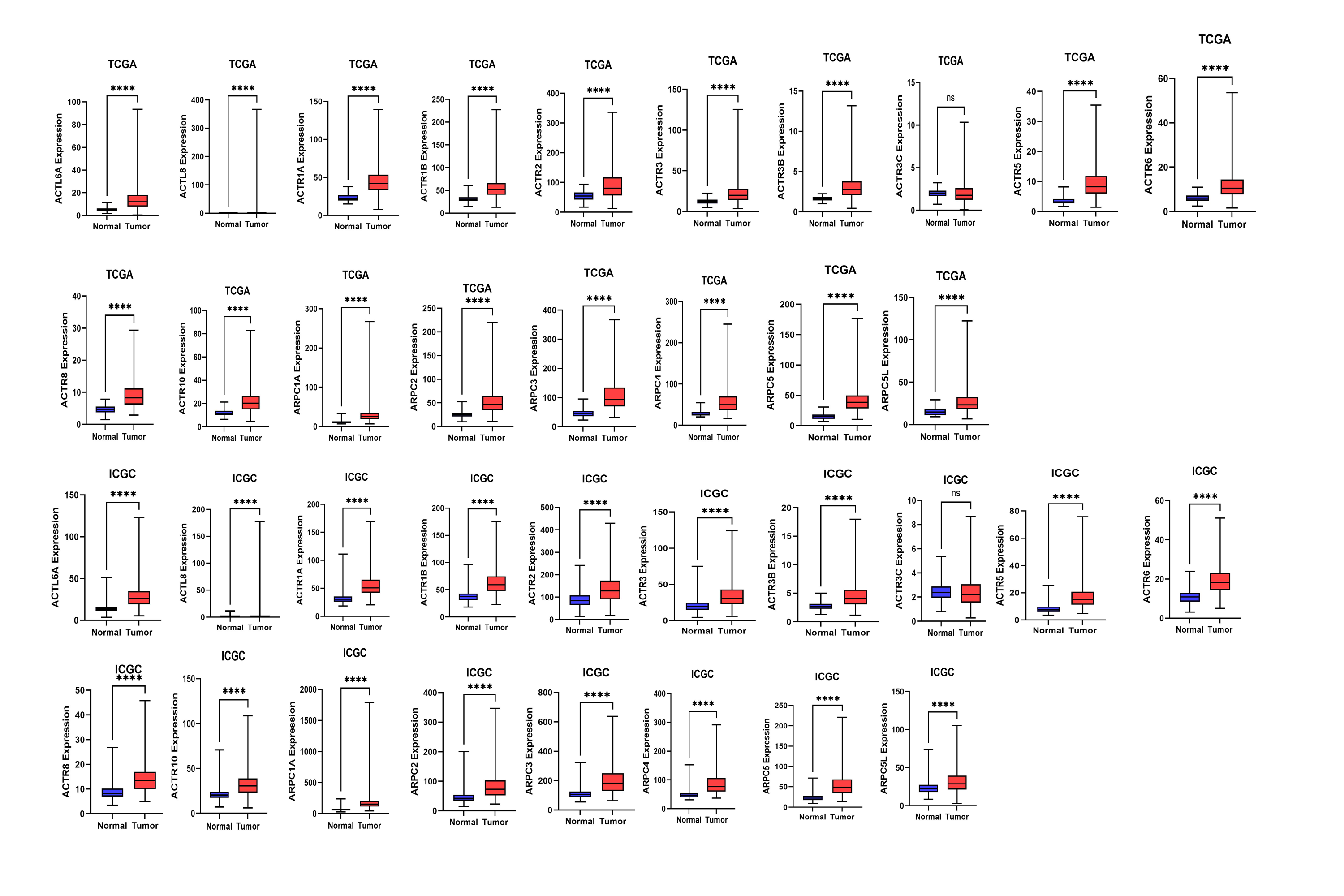

Supplement: Supplementary file 3 [file Image_1.TIF]

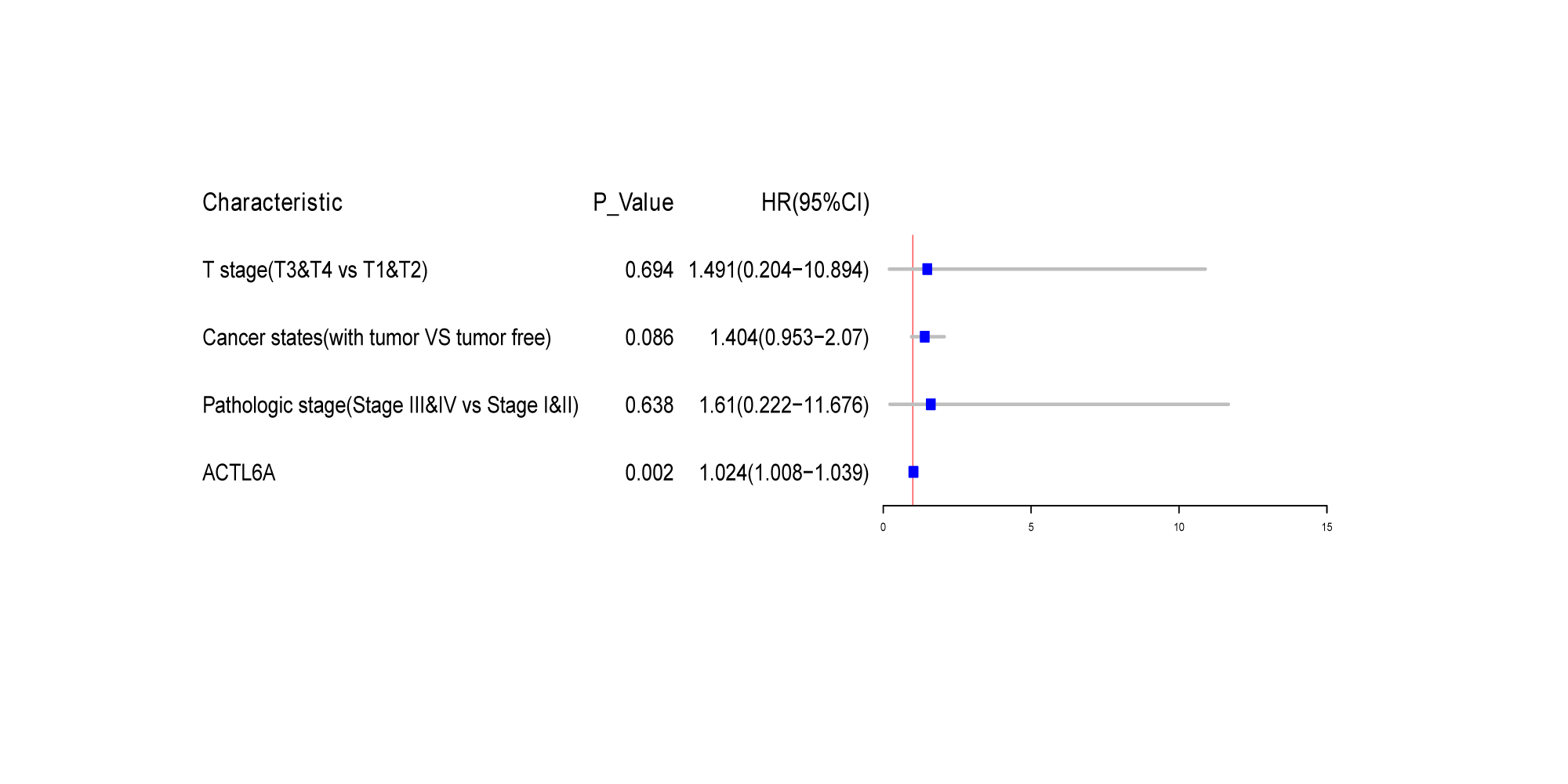

Supplement: Supplementary file 4 [file Image_2.TIF]
